# Nanoparticle-enhanced apigenin suppresses androgen receptor signaling and cellular plasticity in LNCaP cells

**DOI:** 10.1038/s41598-026-63769-5

**Published:** 2026-07-23

**Authors:** Sinan Vicil, Riza Serttas, Suat Erdogan

**Affiliations:** 1 Faculty of Veterinary Medicine, Department of Biochemistry, Tekirdag Namik Kemal University, Tekirdag, Türkiye; 2 School of Medicine, Department of Medical Biology, Trakya University, Edirne, Türkiye

**Keywords:** Apigenin, Selenium nanoparticles, Androgen receptor, Prostate cancer, Apoptosis, Cellular plasticity, Biochemistry, Cancer, Cell biology

## Abstract

Prostate cancer remains highly dependent on androgen receptor (AR) signaling, yet therapeutic escape often emerges through incomplete suppression of AR-driven transcription and activation of compensatory survival pathways. Here, we investigated whether a hyaluronic acid-coated selenium nanoparticle formulation loaded with apigenin (HA-SeNP-Api) could enhance the anti-tumor activity of apigenin in androgen-responsive LNCaP prostate cancer cells. Selenium nanoparticles were generated by reduction of sodium selenite with ascorbic acid, followed by apigenin loading and hyaluronic acid coating. After 72 h treatment, HA-SeNP-Api produced a consistent biological response across several measured endpoints among the tested groups. Apigenin screening identified 6.25 µM as a biologically active sub-IC50 working concentration. At this dose, the nanoformulation reduced AR and PSA mRNA expression to 0.27-fold and 0.42-fold, respectively, and lowered PSA protein to 10% of control. Total apoptosis increased to 44.30%, compared with 31.36% for selenium nanoparticles alone, 9.77% for apigenin, and 3.70% for enzalutamide. The formulation also increased p53 and p21 expression, reduced SOX2, OCT3/4 and SLUG, and retained growth-inhibitory activity in 3D spheroids. These findings suggest that HA-SeNP-Api enhances the biological activity of apigenin under the tested in vitro conditions and modulates AR/PSA-associated readouts, apoptosis-associated responses, and selected plasticity-associated transcripts in LNCaP cells.

**Supplementary Information:**

The online version contains supplementary material available at https://doi.org/10.1038/s41598-026-63769-5.

## Introduction

Prostate cancer remains one of the most frequently diagnosed malignancies in men and a major cause of cancer-related mortality worldwide^1^. One of the defining biological features of this disease is its strong dependence on androgen receptor (AR) signaling, particularly in early and hormone-responsive stages^2^. Although androgen deprivation and second-generation antiandrogen therapies initially provide substantial benefit, disease progression commonly emerges through the adaptive maintenance, reorganization, or partial reactivation of AR-associated transcriptional programs^3,4^. In AR-responsive models such as LNCaP cells, AR signaling therefore functions not only as an initiating driver but also as a persistent determinant of cellular proliferation, metabolic continuity, and treatment adaptation^5,6^.

Another major therapeutic challenge is that AR inhibition alone is often insufficient to achieve durable tumor control. In prostate cancer cells, AR-regulated survival is buffered by cross-talk with compensatory pathways, including PI3K/Akt and other pro-survival signaling nodes, which can preserve viability even when canonical androgen signaling is weakened. This redundancy contributes to reduced treatment sensitivity, incomplete execution of apoptosis, and sustained cell-cycle progression under pharmacological stress^7–9^. Accordingly, interventions that combine suppression of AR output with induction of broader cytotoxic stress may be more effective than single-target antiandrogen strategies alone.

Apigenin is a naturally occurring dietary flavonoid that has attracted attention because of its antiproliferative, pro-apoptotic, anti-inflammatory, and signaling-modulatory properties^10^. In AR-positive prostate cancer models, apigenin has been associated with reduced AR abundance, inhibition of AR nuclear translocation, suppression of PSA transcription, and modulation of cell-cycle regulators and apoptotic mediators^11,12^. These properties make apigenin particularly attractive in a natural product-based therapeutic context, because they link a natural product-based intervention to mechanistically relevant oncogenic pathways. However, despite these advantages, apigenin suffers from poor aqueous solubility and limited bioavailability, both of which may restrict intracellular delivery and reduce its practical therapeutic efficacy when used alone^13,14^.

Nanotechnology-based delivery systems provide a rational strategy to overcome these limitations^15^. Because oxidative balance is closely linked to cancer cell survival and treatment response, redox-modulating platforms may offer additional therapeutic advantages in oncology^16,17^. Selenium nanoparticles (SeNPs) are of particular interest in oncology because they can disrupt redox balance, modulate intracellular signaling, promote apoptosis, and at the same time serve as functional carrier platforms. In prostate cancer models, selenium-based approaches have been reported not only to enhance anti-proliferative responses but also to interfere with AR-related signaling^18–20^. Hyaluronic acid (HA), in turn, offers a potentially useful biological interface in tumor systems expressing CD44^21,22^. CD44 expression and isoform switching have been linked to cancer stem cell state and disease progression in multiple tumor types^23,24^. However, in the present study, HA coating was not used to support a direct claim of receptor-mediated selectivity; rather, it was incorporated as a functional surface component that could improve cell-associated interaction and formulation performance in tumor cells. This distinction is important because HA/CD44 biology is context-dependent and should be interpreted cautiously in cancer models^25,26^.

A companion study from our group has separately reported the physicochemical characterization of the HA-coated apigenin-loaded selenium nanoparticle platform used in the present work—including particle size, zeta potential, encapsulation efficiency, and in vitro release profile—together with its activity against the CD44⁺ prostate cancer stem cell subpopulation^27^. To avoid extensive overlap with that work, the present manuscript focuses on a mechanistically distinct biological investigation in the parental androgen-responsive LNCaP population, while the key previously reported physicochemical properties of the HA-SeNP-Api formulation are summarized in Supplementary Table S2 for formulation context. The current study specifically emphasizes AR/PSA-axis readouts, apoptotic and checkpoint-associated responses, cell-cycle redistribution, and plasticity-associated transcriptional markers—endpoints that were not addressed in the companion study.

Importantly, the present work was not designed to rediscover the known anticancer activity of apigenin. Rather, it was designed to determine whether incorporation of a low, biologically active apigenin concentration into a HA-coated selenium nanoparticle platform could reshape androgen-responsive signaling and stress-response biology in the parental LNCaP population. This distinguishes the present study from our companion work on the CD44⁺ prostate cancer stem cell subpopulation, because the current manuscript focuses on AR/PSA-axis readouts, PSA protein output, apoptosis-associated responses, cell-cycle redistribution, and plasticity-associated transcriptional responses in androgen-responsive LNCaP cells.

Based on this rationale, the present study was designed to investigate whether apigenin-loaded HA-coated selenium nanoparticles could produce a more consistent biological response than free apigenin, enzalutamide, or SeNPs alone across selected molecular and phenotypic endpoints in LNCaP cells. The study specifically focused on four measured response layers: AR/PSA-axis readouts, apoptosis-associated responses, cell-cycle distribution, and stemness/plasticity-associated transcriptional markers. In addition to this mechanistic evaluation, the study also aimed to identify a biologically active sub-IC_50_ apigenin concentration and to determine whether HA coating could functionally enhance the cytotoxic effect of the selenium-based nanostructure while preserving a more acceptable preliminary tolerability profile in ARPE19 cells.

## Results

Apigenin viability screening identified 6.25 µM as a biologically active working concentration, and HA coating improved the functional performance of the selenium-based formulation.

**Fig. 1:**
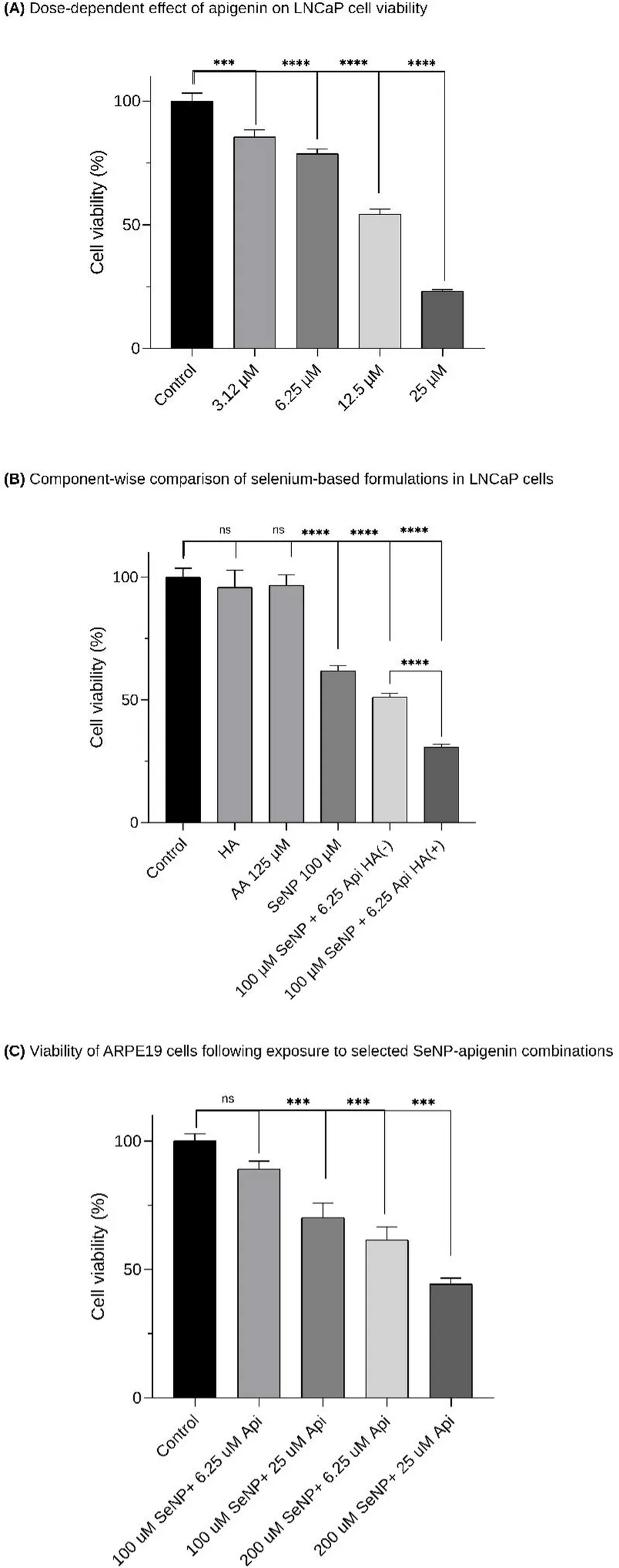
Preliminary viability screening and selection of the working HA-SeNP-Api formulation. (**A**) Dose-dependent effect of apigenin on LNCaP cell viability. Cells were exposed to increasing concentrations of apigenin (3.12, 6.25, 12.5, and 25 µM), and viability was assessed by MTT assay. Apigenin reduced viability in a dose-dependent manner, and 6.25 µM was selected as a biologically active sub-IC_50_ concentration for subsequent mechanistic analyses. (**B**) Component-wise comparison demonstrated that AA (125 µM) and HA alone did not significantly affect LNCaP viability relative to the control group (*P* > 0.05), whereas SeNP treatment significantly reduced viability (*P* < 0.0001). Loading apigenin onto SeNPs further enhanced the cytotoxic response, as the HA(−) formulation produced a significantly greater reduction in viability than SeNP alone (*P* = 0.0058). HA coating was associated with a further reduction in viability, with the HA(+) formulation showing significantly lower viability than both SeNP alone and HA(−) (both *P* < 0.0001). Overall, these findings showed a stepwise pattern of reduced viability across selenium-containing formulations, with HA-SeNP-Api [HA(+)] showing the lowest LNCaP viability under the tested conditions. (**C**) Viability of ARPE19 cells following exposure to selected SeNP-apigenin combinations. Cells were treated with 100 µM SeNP + 6.25 µM apigenin, 100 µM SeNP + 25 µM apigenin, 200 µM SeNP + 6.25 µM apigenin, or 200 µM SeNP + 25 µM apigenin. The lower-dose combination showed the mildest reduction in ARPE19 viability, whereas higher-dose combinations caused greater viability loss. Values are presented as mean ± SEM; *n* = 5 independent biological replicates. Statistical analysis was performed using Brown-Forsythe and Welch ANOVA followed by Games-Howell or Dunnett’s T3 multiple-comparisons tests, as appropriate. Different letters or significance markers indicate statistically significant differences at *P* < 0.05.

Initial MTT screening showed that apigenin reduced the viability of LNCaP cells in a dose-dependent manner across the tested concentration range (Fig. 1A). Based on this response profile, 6.25 µM was selected as a biologically active sub-IC_50_ concentration for subsequent mechanistic analyses. Component-wise comparison demonstrated that AA (125 µM) and HA alone did not significantly affect LNCaP viability relative to the control group (*P* > 0.05), whereas SeNP treatment significantly reduced viability (*P* < 0.0001). Loading apigenin onto SeNPs further enhanced the cytotoxic response, as the HA(−) formulation produced a significantly greater reduction in viability than SeNP alone (*P* = 0.0058). HA coating was associated with a further reduction in viability, with the HA(+) formulation showing significantly lower viability than SeNP alone and HA(−) (both *P* < 0.0001). Overall, these findings showed a stepwise pattern of reduced viability across selenium-containing formulations, with HA-SeNP-Api [HA(+)] showing the lowest LNCaP viability under the tested conditions (Fig. 1B). In parallel, preliminary tolerability assessment in ARPE19 cells indicated that the lower-dose SeNP-apigenin combination showed the mildest reduction in non-tumoral cell viability, whereas higher-dose combinations produced a more pronounced loss of viability (Fig. 1C). Together, these findings supported the use of the lower-dose HA-SeNP-Api configuration in downstream experiments as a biologically active and comparatively more acceptable working condition.

### HA-SeNP-Api attenuated AR/PSA-axis readouts in LNCaP cells

**Fig. 2:**
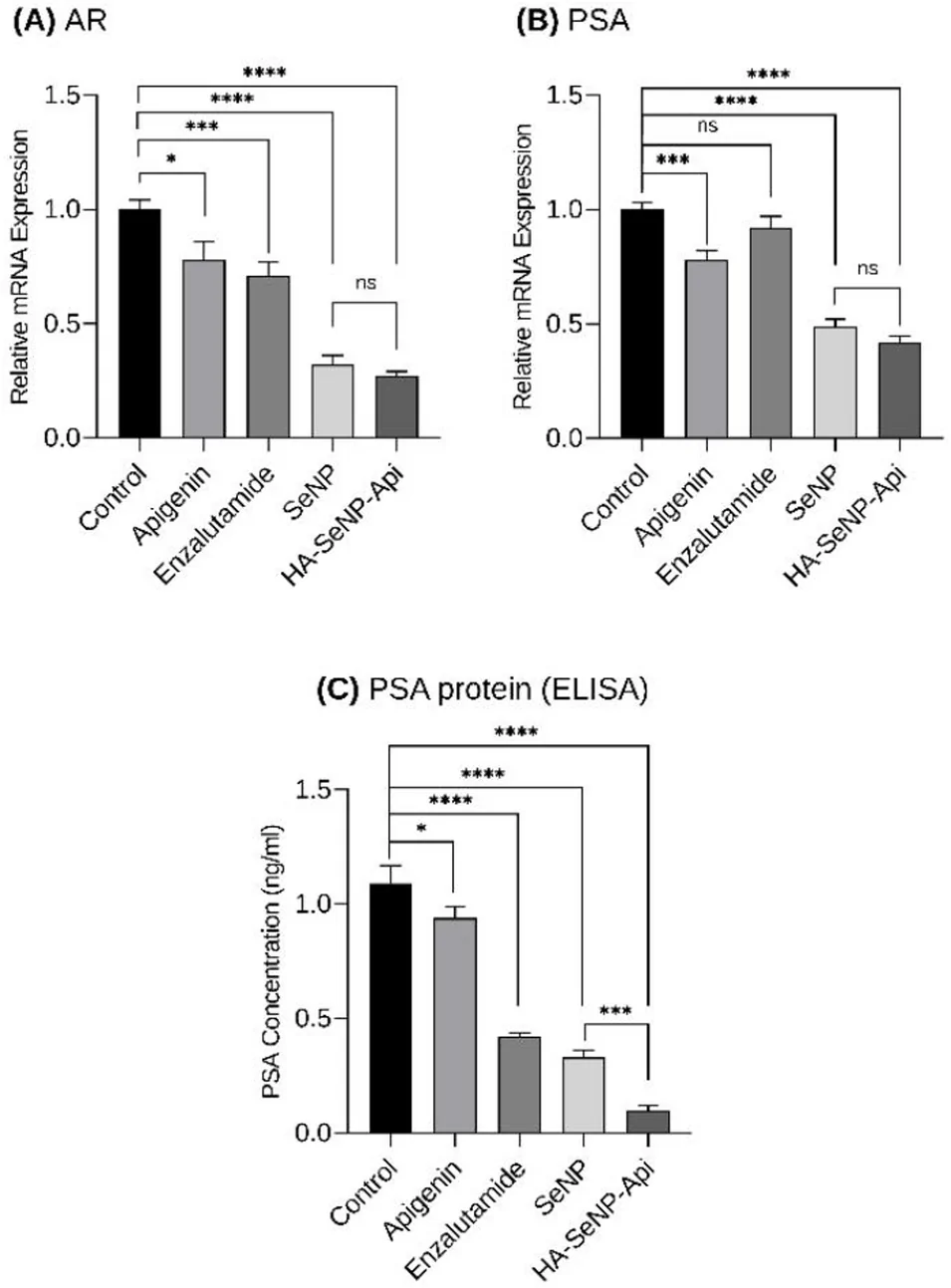
HA-SeNP-Api attenuates measured AR/PSA-axis readouts in LNCaP cells. (**A**) Relative AR mRNA expression, (**B**) Relative PSA mRNA expression, (**C**) PSA protein levels determined by ELISA. LNCaP cells were treated for 72 h with apigenin (6.25 µM), enzalutamide (6.25 µM), SeNP (100 µM), or HA-SeNP-Api (100 µM SeNP + 6.25 µM apigenin + 0.1% HA). RT-qPCR data are shown as mean ± SEM from *n* = 3 independent biological replicates; ELISA data represent *n* = 3 independent biological replicates. Statistical analysis was performed using one-way ANOVA followed by Tukey’s multiple-comparisons test for panels (**A**) and (**C**), and Sidak’s multiple-comparisons test for panel (**B**). Statistical significance was accepted at *P* < 0.05.

To determine whether the selected formulation affected androgen-responsive signaling, AR and PSA expression were evaluated at both transcript and protein levels following 72 h treatment (Fig. 2A–C). Among the tested groups, HA-SeNP-Api produced the most consistent reduction across the measured AR/PSA-axis readouts, reducing AR mRNA expression to 0.27-fold, PSA mRNA expression to 0.42-fold, and PSA protein level to 0.10 relative units. Apigenin alone also reduced PSA mRNA under the present experimental conditions. However, this comparison should be interpreted cautiously because enzalutamide exposure was not optimized to establish a pharmacological potency comparison. These results indicate that the HA-coated selenium nanoparticle platform was associated with a broader reduction in the measured AR/PSA-axis readouts than free apigenin under the tested conditions (Fig. 2A–C).

### HA-SeNP-Api increased annexin V/PI-defined apoptotic populations and apoptosis-associated transcripts

**Fig. 3:**
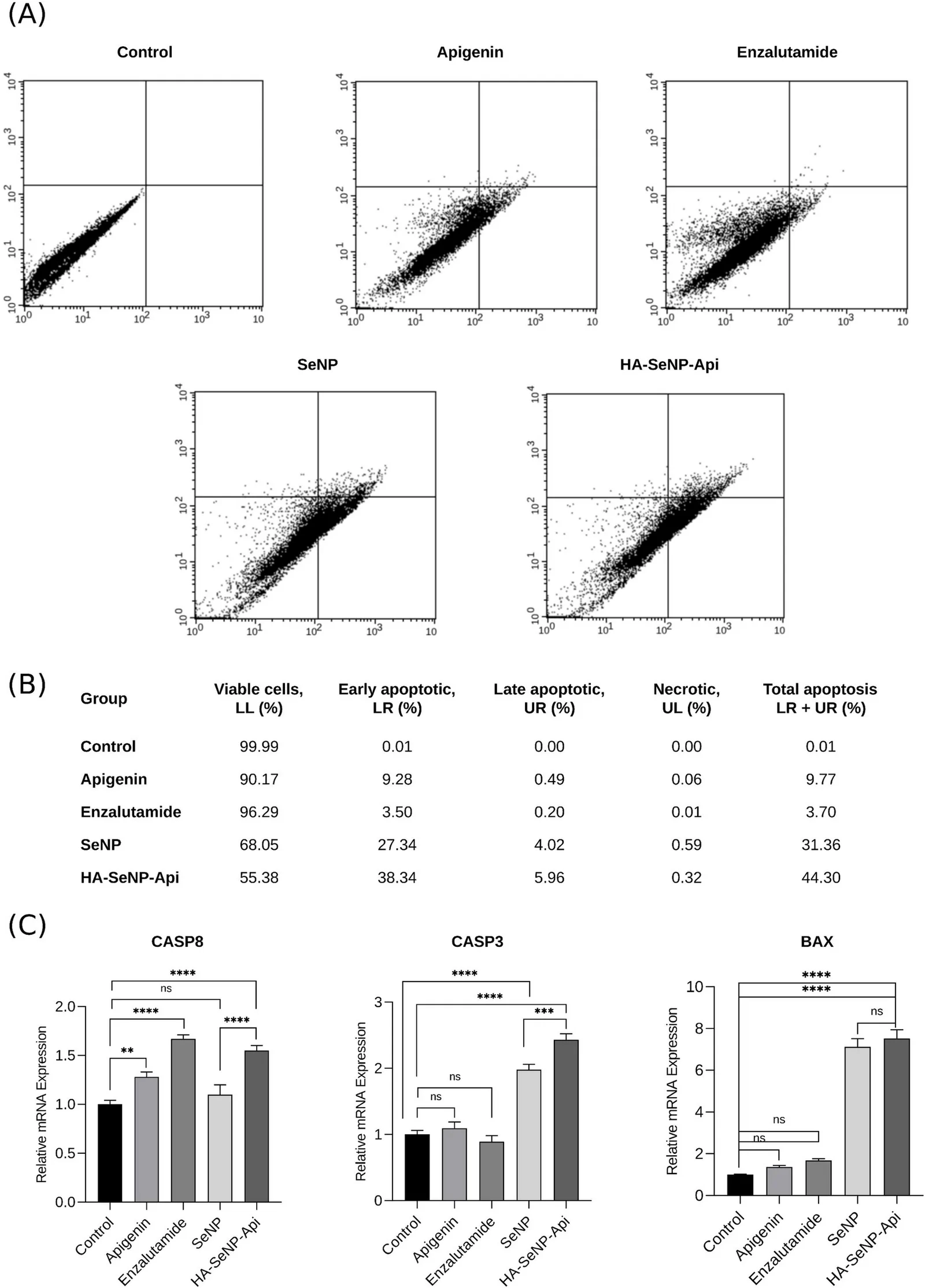
HA-SeNP-Api increases Annexin V/PI-defined apoptotic populations in LNCaP cells. (**A**) Representative Annexin V-FITC/propidium iodide (PI) flow cytometry quadrant plots following 72 h treatment. (**B**) Quadrant-based apoptosis distribution. LL, viable cells; LR, early apoptotic cells; UR, late apoptotic cells; UL, necrotic/damaged cells. Total apoptosis was calculated as LR + UR. (**C**) Relative mRNA expression levels of apoptosis-associated genes CASP8, CASP3, and BAX. Data are presented as mean ± SEM from *n* = 3 independent biological replicates. Statistical analysis was performed using one-way ANOVA followed by Tukey’s multiple-comparisons test. Statistical significance was accepted at *P* < 0.05.

**Fig. 4:**
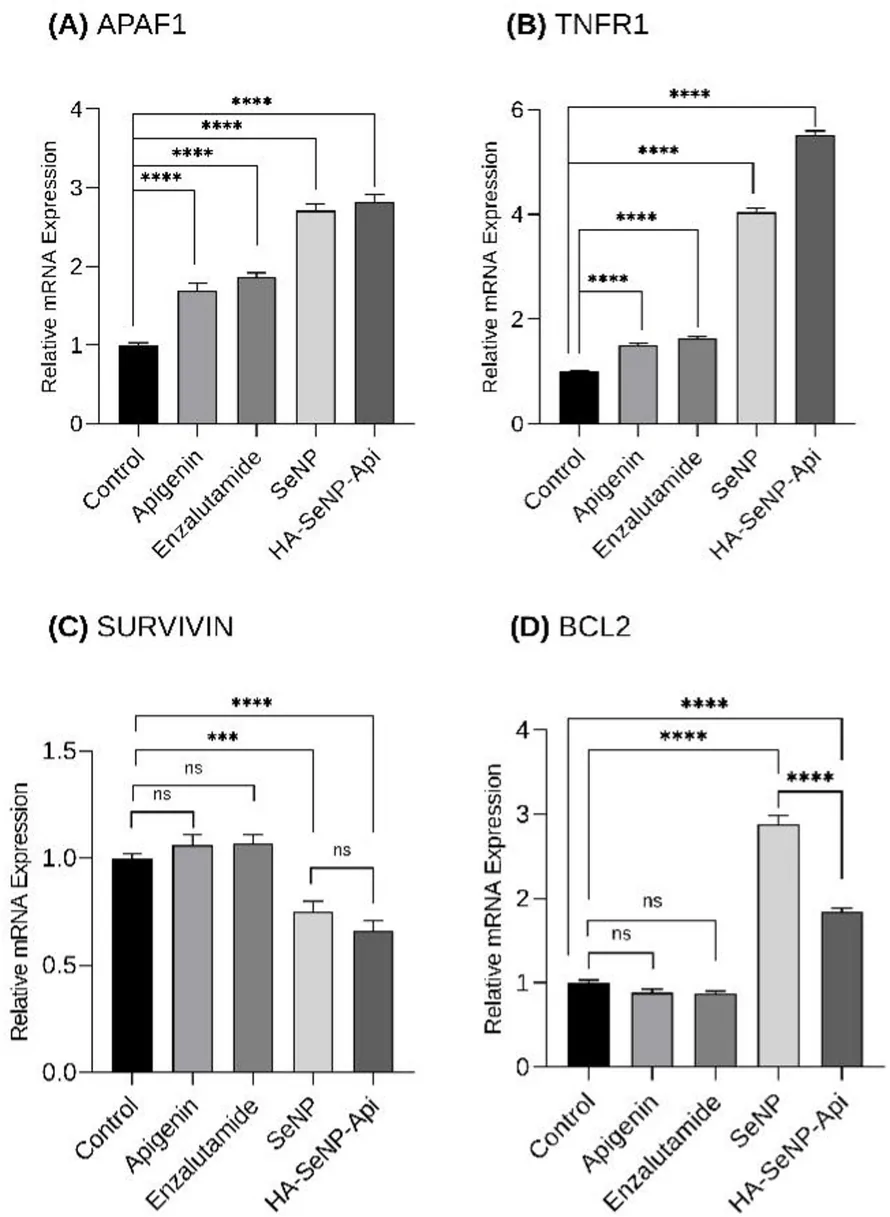
Relative mRNA expression levels of additional apoptosis- and survival-related genes in LNCaP cells following treatment. (**A**) APAF1, (**B**) TNFR1, (**C**) Survivin, and (**D**) BCL2. LNCaP cells were treated for 72 h with apigenin (6.25 µM), enzalutamide (6.25 µM), SeNP (100 µM), or HA-SeNP-Api (100 µM SeNP + 6.25 µM apigenin + 0.1% HA). Data are presented as mean ± SEM from *n* = 3 independent biological replicates. Statistical analysis was performed using one-way ANOVA followed by Tukey’s multiple-comparisons test. Statistical significance was accepted at *P* < 0.05.

Annexin V/PI flow cytometric analysis indicated a treatment-associated increase in apoptotic populations, with the highest total apoptosis value observed in the HA-SeNP-Api group (Fig. 3A, B). Quantitative analysis showed that total apoptosis reached 44.30% in the HA-SeNP-Api group, compared with 31.36% in the SeNP group, 9.77% in the apigenin group, and 3.70% in the enzalutamide group. This flow cytometric pattern was supported by transcriptional changes in apoptosis-associated genes. As shown in Fig. 3C, HA-SeNP-Api increased the expression of CASP8, CASP3, and BAX relative to the control group, consistent with activation of apoptotic signaling. Supplementary analyses further extended this pattern to additional apoptosis- and survival-related markers, including APAF1, TNFR1, survivin, and BCL2 (Fig. 4). Collectively, these findings support a stronger apoptosis-associated response in the HA-SeNP-Api group than in the free apigenin or enzalutamide groups under the tested conditions. Although BCL2 expression was increased in selenium-containing treatments, the overall Annexin V/PI profile remained consistent with increased apoptosis. In this context, the increase in BCL2 appears to reflect an inadequate compensatory anti-apoptotic response rather than effective cell death protection.

### HA-SeNP-Api was associated with cell-cycle redistribution and checkpoint-associated transcriptional changes

**Fig. 5:**
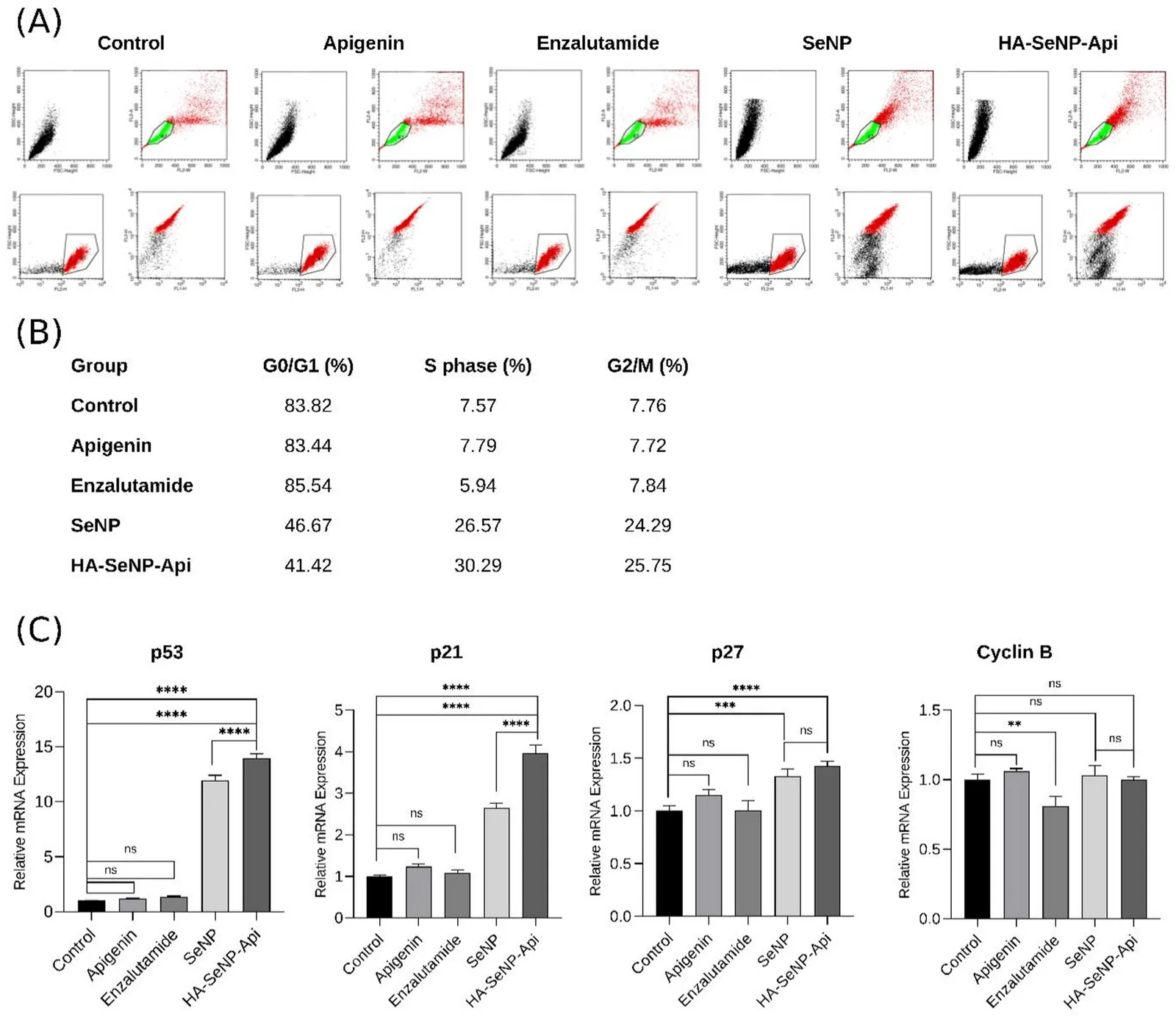
HA-SeNP-Api is associated with cell-cycle redistribution and checkpoint-associated transcriptional changes in LNCaP cells. (**A**) Representative PI-based flow cytometry plots of cell-cycle distribution in the control, apigenin, enzalutamide, SeNP, and HA-SeNP-Api groups after 72 h of treatment. (**B**) Quantitative distribution of cells in G0/G1, S, and G2/M phases. (**C**) Relative mRNA expression levels of checkpoint-associated genes p53, p21, p27, and Cyclin B. Flow cytometry data in panels A and B are shown as representative plots and corresponding descriptive summary values, respectively. RT-qPCR data in panel C are presented as mean ± SEM from *n* = 3 independent biological replicates. Statistical analysis for panel C was performed using one-way ANOVA followed by Tukey’s multiple-comparisons test. Statistical significance was accepted at *P* < 0.05.

Cell-cycle analysis demonstrated that treatment with HA-SeNP-Api altered the phase distribution of LNCaP cells relative to the control condition (Fig. 5A, B). Among the tested groups, the nanoformulation showed a marked redistribution of cell-cycle phases, consistent with altered cell-cycle progression. This shift was accompanied by transcriptional activation of checkpoint-associated genes, as shown by the increased expression of p53 and p21 in the HA-SeNP-Api group (Fig. 5C). Changes in additional checkpoint-related markers, including p27 and Cyclin B, were also evaluated within the same dataset. Taken together, the flow cytometric and transcriptional findings support the conclusion that HA-SeNP-Api not only reduced androgen-responsive signaling and promoted apoptosis, but also was associated with checkpoint-related transcriptional changes and altered cell-cycle distribution in LNCaP cells.

### HA-SeNP-Api reduced selected plasticity-associated transcripts

**Fig. 6:**
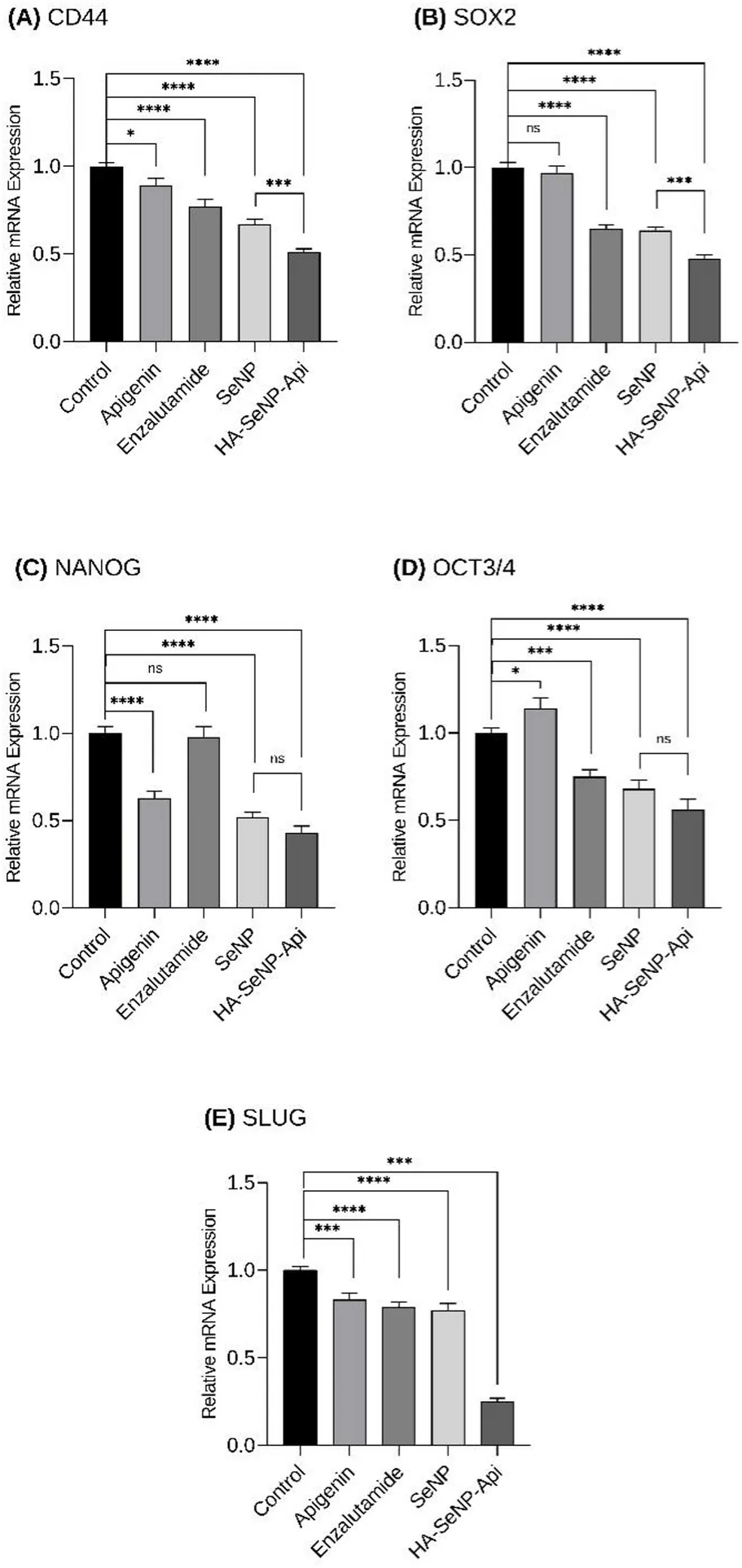
HA-SeNP-Api reduces selected plasticity-associated transcripts in LNCaP cells. Relative mRNA expression levels of (**A**) CD44, (**B**) SOX2, (**C**) NANOG, (**D**) OCT3/4, and (**E**) SLUG in LNCaP cells after 72 h of treatment with apigenin (6.25 µM), enzalutamide (6.25 µM), SeNP (100 µM), or HA-SeNP-Api (100 µM SeNP + 6.25 µM apigenin + 0.1% HA). Data are presented as mean ± SEM from *n* = 3 independent biological replicates. Statistical analysis was performed using one-way ANOVA followed by Tukey’s multiple-comparisons test. Statistical significance was accepted at *P* < 0.05.

To examine whether the biological response extended beyond androgen signaling and apoptosis, transcripts associated with stemness and cellular plasticity were analyzed. As shown in Fig. 6, treatment with HA-SeNP-Api reduced the expression of multiple plasticity-associated markers, including CD44, SOX2, NANOG, OCT3/4, and SLUG. Although treatment-related changes were also detectable in some comparator groups, the most consistent overall attenuation across the analyzed transcriptional markers was observed in the HA-SeNP-Api group. These findings indicate that the selected nanoformulation reduced selected transcripts associated with phenotypic flexibility in LNCaP cells.

### Growth-inhibitory activity of HA-SeNP-Api was preserved under 3D spheroid conditions

**Fig. 7:**
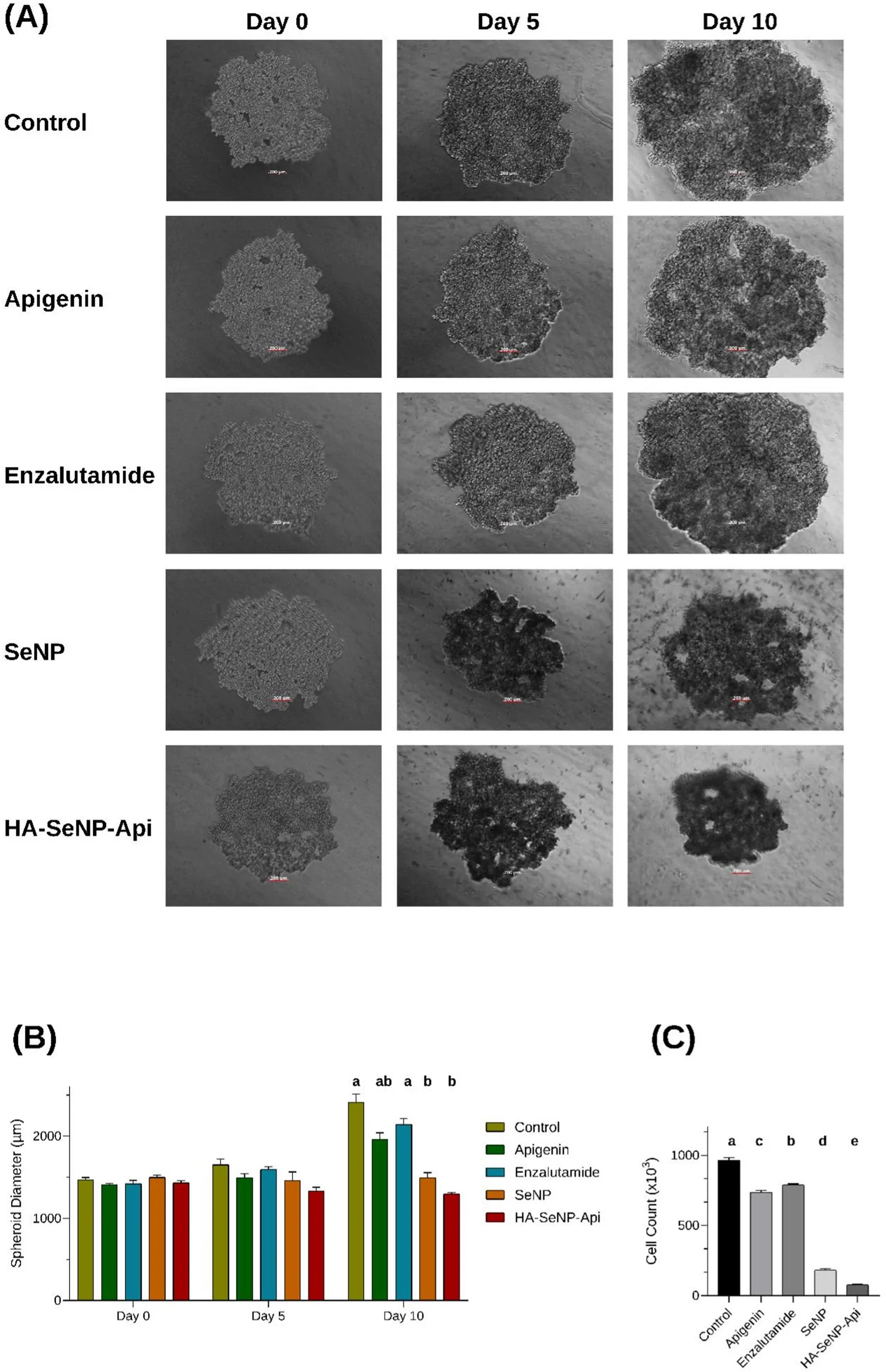
Selenium-containing treatments reduce 3D spheroid growth and endpoint cell number in LNCaP cells. (**A**) Representative bright-field images of LNCaP spheroids in the Control, Apigenin, Enzalutamide, SeNP, and HA-SeNP-Api groups at Day 0, Day 5, and Day 10. Scale bar: 200 μm. (**B**) Changes in spheroid diameter over time. Spheroid diameter data obtained from the same spheroids at Day 0, Day 5, and Day 10 were analyzed by two-way repeated-measures ANOVA with Geisser-Greenhouse correction followed by Tukey’s multiple-comparisons test. By Day 10, spheroid diameter was significantly reduced in the SeNP and HA-SeNP-Api groups compared with the Control group, whereas no significant difference was detected between SeNP and HA-SeNP-Api at Day 10. (**C**) Final spheroid cell counts at the experimental endpoint. Groups were compared by one-way ANOVA followed by Tukey’s multiple-comparisons test. Different letters indicate statistically significant differences between groups (*P* < 0.05). Data are presented as mean ± SEM from *n* = 3 independent biological replicates. Statistical significance was accepted at *P* < 0.05.

The antitumor activity of the formulation was further evaluated in a three-dimensional spheroid model (Fig. 7A–C). Two-way repeated measures ANOVA revealed significant effects of time, treatment, and time × treatment interaction on spheroid diameter (all *P* < 0.0001), indicating that treatment responses diverged over time. No significant differences among groups were detected at Day 0 or Day 5. However, by Day 10, spheroid diameter was significantly lower in the SeNP and HA-SeNP-Api groups compared with both the control group (*P* = 0.0366 and *P* = 0.0441, respectively) and the enzalutamide group (*P* = 0.0351 and *P* = 0.0323, respectively), whereas no significant difference was detected between SeNP and HA-SeNP-Api (*P* = 0.4445). Consistent with these findings, selenium-containing treatments also showed reduced final spheroid cellular burden at the endpoint (Fig. 7C). Together, these data indicate that growth-inhibitory activity was preserved under 3D culture conditions, with reduced spheroid growth observed mainly in the selenium-containing groups.

**Fig. 8:**
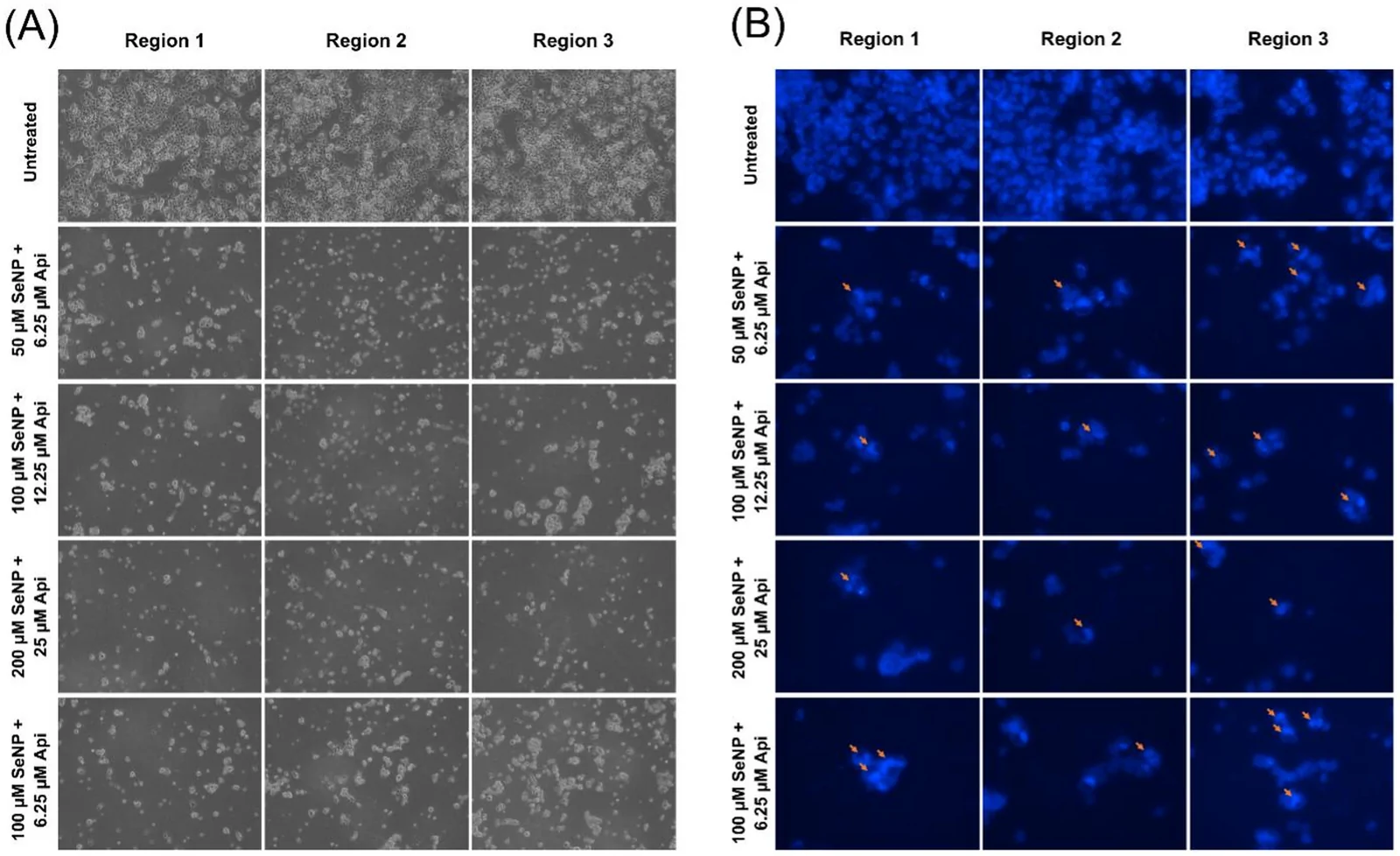
Representative qualitative morphological observations following SeNP-apigenin treatments in LNCaP cells. (**A**) Representative bright-field images obtained using a 10× objective, showing treatment-associated changes in cell density, rounding, and detachment-like morphology. (**B**) Representative fluorescence images obtained using a 40× objective, showing nuclear morphology. Orange arrows indicate cells with apoptotic-like nuclear condensation or fragmentation. Bright-field and fluorescence images were selected from representative fields of the corresponding treatment groups and were not intended to represent identical matched fields of view. These images were used as qualitative morphological support; quantitative apoptosis assessment was based on Annexin V/PI flow cytometry.

### Treatment-associated morphological changes supported the cytotoxic profile of selenium-apigenin combinations

Representative bright-field and fluorescence images provided qualitative morphological support for the cytotoxic and apoptosis-associated profile observed in the viability and Annexin V/PI assays (Fig 8). In bright-field images obtained using a 10× objective, selenium-apigenin-treated cells showed reduced cell density, increased rounding, and detachment-like changes compared with untreated cells. Fluorescence images obtained using a 40× objective showed occasional apoptotic-like nuclear condensation or fragmentation, indicated by arrows. Because these images were obtained as representative morphological observations rather than as a quantitative imaging assay, they were interpreted as supportive visual evidence and not as an independent demonstration of a dose-dependent apoptotic response.

## Discussion

The novelty of the present study does not lie in demonstrating that apigenin can affect prostate cancer cells, as this has been reported previously. Instead, the contribution of this work is the evaluation of a HA-coated selenium nanoparticle-based apigenin formulation in parental androgen-responsive LNCaP cells, with integrated assessment of AR/PSA output, apoptosis, checkpoint-associated transcription, cell-cycle redistribution, plasticity-associated transcripts, and 3D spheroid growth.

The present study suggests that an apigenin-loaded, hyaluronic acid-coated selenium nanoparticle formulation produces a coordinated in vitro response across several measured endpoints in androgen-responsive LNCaP prostate cancer cells. Among the evaluated endpoints, attenuation of AR/PSA-axis readouts emerged as one of the most consistent molecular responses, accompanied by increased Annexin V/PI-defined apoptotic populations, checkpoint-associated transcriptional changes, altered cell-cycle distribution, and reduced expression of selected plasticity-associated transcripts. Within the scope of this AR-responsive model, these findings support the interpretation that the formulation affected multiple measured response layers rather than only reducing cell viability. This interpretation is consistent with the study rationale established in the manuscript, in which AR-associated signaling, apoptosis-related readouts, cell-cycle distribution, and plasticity-related transcription were evaluated as interconnected but experimentally distinct endpoints.

One of the central findings of the study is the reduction in measured AR/PSA-axis readouts. In prostate cancer, AR activity continues to serve as a central survival and progression axis even when therapeutic pressure is applied, and disease persistence is often associated with incomplete suppression of androgen-responsive transcriptional output. In this context, the reduction of AR mRNA, PSA mRNA, and PSA protein observed in the HA-SeNP-Api group suggests that the formulation affected androgen-responsive molecular readouts in addition to reducing cell viability. This interpretation is also compatible with earlier reports cited in the draft showing that apigenin can reduce AR abundance and suppress PSA transcription in AR-positive prostate cancer models^11^. The present findings extend that literature by indicating that apigenin retains this antiandrogenic orientation when incorporated into the selenium-based HA-coated nanosystem, and that the composite formulation produced a broader reduction in the measured AR/PSA-related endpoints than the free compound under the same experimental conditions.

Free apigenin also reduced PSA mRNA under the present experimental conditions, but this observation should be interpreted cautiously because the study was not designed to compare the pharmacological potency of apigenin and enzalutamide. Rather than implying superiority over a clinically established antiandrogen in a broad translational sense, the result more reasonably suggests that apigenin may influence androgen-responsive transcription through partially overlapping but not identical regulatory routes within LNCaP cells^28^. This possibility is compatible with the literature framework already cited in the manuscript, where apigenin is described not only as an antiproliferative flavonoid but also as a modulator of AR-linked signaling and PSA-related transcriptional output^29^.

The pro-apoptotic findings further strengthen this integrated interpretation. Total apoptosis increased stepwise across the treatment groups and reached its highest level in the HA-SeNP-Api condition, suggesting that the formulation was associated with increased apoptosis-related responses in addition to changes in androgen-related transcriptional readouts. This pattern was supported by the increased expression of pro-apoptotic markers such as BAX, CASP3, APAF1, and TNFR1, together with reduced survival-associated signaling. Earlier studies cited in the manuscript already frame apigenin as a compound capable of modulating apoptotic mediators in prostate cancer models, while selenium nanoparticles are discussed as redox-active and apoptosis-promoting oncologic platforms. The current data are therefore consistent with both arms of that literature base: the phytotherapeutic component contributes a biologically relevant pro-apoptotic signal, and the selenium-based component may have contributed to the stronger apoptosis-associated profile observed under the tested conditions.

Checkpoint- and survival-related transcripts further supported this interpretation. In particular, the marked increase in p53 expression in the selenium-containing groups, together with the reduction in survivin expression, supported the interpretation that selenium-containing treatments were associated with an apoptosis-oriented transcriptional profile. Although survivin remained close to baseline in the apigenin and enzalutamide groups, it declined clearly in the SeNP and HA-SeNP-Api groups, supporting the idea that selenium-based treatment contributed to weakening anti-apoptotic protection. At the same time, although BCL2 expression increased in selenium-containing treatments, the Annexin V/PI profile remained consistent with increased apoptotic populations. In this context, the increase in BCL2 appears to reflect an inadequate compensatory anti-apoptotic response rather than effective protection from cell death. Thus, BCL2 upregulation may reflect a stress-induced compensatory transcriptional response that remains insufficient to prevent apoptosis once the pro-apoptotic threshold is exceeded^32^.

The observed response may reflect the contribution of three formulation-related elements. First, apigenin provides a pharmacological component previously associated with AR-related and apoptosis-related effects. Second, selenium nanoparticles may contribute a redox- and stress-associated component and may also contribute to weakening AR-associated survival signaling, as suggested by prior prostate cancer-oriented selenium literature cited in the draft^18,19^. Third, the hyaluronic acid coating may improve cell-associated interaction and functional exposure of the loaded cargo^30,31^.

The component-wise viability comparison further clarified the functional contribution of each formulation element. Neither AA nor HA alone significantly affected LNCaP viability, indicating that the marked cytotoxic response was not driven by these components in isolation. In contrast, selenium-containing treatments produced a clear stepwise increase in growth inhibition, with additional suppression after apigenin loading and a further significant enhancement after HA coating. This pattern is consistent with the possibility that HA coating contributed to improved formulation performance under the present experimental conditions.

Importantly, however, the present results should not be overstated as direct proof of receptor-mediated targeting, because the current study did not include a formal mechanistic validation of CD44-dependent uptake. This more conservative interpretation is fully aligned with the conceptual position already stated in the manuscript, namely that HA was used here as a functional surface component rather than as the basis for a definitive selectivity claim.

The cell-cycle and checkpoint-related findings are also biologically important. Increased p53 and p21 expression, together with treatment-associated redistribution of cell-cycle phases, suggest that HA-SeNP-Api was associated with checkpoint-related transcriptional changes in addition to reduced viability. In prostate cancer cells, incomplete apoptosis is often accompanied by continued cycling or partial checkpoint adaptation^32^. Therefore, the concurrent appearance of enhanced apoptosis and checkpoint-associated transcription supports the idea that the formulation affected multiple measured response layers. This is conceptually consistent with the rationale established in the Introduction that AR-directed suppression alone is frequently insufficient and that broader cytotoxic stress may be required to disrupt compensatory survival behavior in AR-responsive tumor cells^7–9^. The present data fit that framework well: HA-SeNP-Api did not act only as an androgen-axis suppressor, but also as a formulation associated with checkpoint-related transcriptional activation and increased apoptotic populations.

The cell-cycle redistribution pattern observed in the selenium-containing treatment groups warrants careful mechanistic consideration in this context. Flow cytometric analysis revealed a marked reduction in the G0/G1 fraction accompanied by a corresponding increase in S and G2/M phase populations, a pattern that might appear contradictory to the concurrent transcriptional upregulation of p21. However, this apparent inconsistency is reconcilable within the framework of p21’s well-established context-dependent biology. Although p21 is classically regarded as a mediator of G1 arrest through inhibition of CDK4/6–cyclin D and CDK2–cyclin E complexes, it also directly inhibits CDK1–cyclin B1, thereby contributing to restraint of G2/M progression^33,34^. In the setting of strong redox-associated stress, as imposed by selenium nanoparticles, the dominant cell-cycle response may therefore reflect S-phase entry and subsequent G2/M accumulation rather than classical G1 arrest, while p21 induction simultaneously signals checkpoint activation and apoptotic commitment at these later phases^35^. This interpretation is further supported by the observation that Cyclin B levels did not significantly increase despite G2/M accumulation, which may indicate that cells were retained at or before the G2/M boundary rather than actively transiting through mitosis. Collectively, the transcriptional and flow cytometric data are therefore not mutually exclusive but instead suggest that p21 induction in the present context reflects a broader stress-response program — encompassing checkpoint signaling, replication stress, and apoptotic priming — rather than a phase-specific arrest signal operating exclusively at the G1/S boundary.

Another noteworthy aspect of the study is the attenuation of plasticity-associated transcriptional markers, including SOX2, OCT3/4, and SLUG. In the context of LNCaP cells, these readouts should not be interpreted as direct evidence of cancer stem cell eradication. However, they remain biologically meaningful because they suggest reduced expression of selected transcripts associated with phenotypic flexibility and adaptive state transitions. This is particularly relevant in prostate cancer, where treatment resistance is often sustained not only by persistent AR output but also by the capacity of tumor cells to engage broader adaptive regulatory programs under stress^36^. In this regard, studies focusing on CD44-associated targeting in prostate cancer and on multilayered regulatory mechanisms that support tumor adaptability reinforce the value of monitoring such plasticity-related markers, even outside a strictly defined cancer stem cell framework^37,38^. The fact that HA-SeNP-Api affected AR/PSA-related readouts and reduced several plasticity-associated transcripts suggests that the formulation affected both AR/PSA-related readouts and selected plasticity-associated transcripts in the present model. Within the boundaries of the present model, this dual effect adds mechanistic depth to the observed cytotoxic phenotype. Among these, SLUG is particularly noteworthy in the context of prostate cancer progression, as it is a transcriptional repressor associated with epithelial-to-mesenchymal transition and has been linked to invasive behavior and treatment-adaptive phenotypic shifts in tumor cells. Its attenuation in the HA-SeNP-Api group therefore suggests that the formulation may also affect a transcript associated with EMT-related and adaptive phenotypes, although functional migration or invasion assays are required to confirm this interpretation.

The 3D spheroid findings provide additional support for the biological relevance of the formulation. Responses observed only in monolayer systems may overestimate activity because they do not reflect diffusion-related constraints or multicellular structural behavior. In the present study, however, growth-inhibitory activity was preserved under spheroid conditions. This effect became statistically evident at Day 10, when both SeNP and HA-SeNP-Api significantly reduced spheroid diameter relative to control and enzalutamide. Although HA-SeNP-Api exhibited the lowest mean spheroid diameter, its effect did not differ significantly from that of SeNP, suggesting that selenium-containing treatments accounted for the principal anti-spheroid activity under 3D conditions. The comparable activity of SeNP and HA-SeNP-Api in 3D spheroids by Day 10 may reflect diffusion-limited penetration of apigenin-loaded nanoparticles into the spheroid core, a possibility that warrants future investigation using confocal Z-stack imaging. While these data do not substitute for in vivo validation, they indicate that growth-inhibitory activity was retained under 3D culture conditions. Within a phytotherapy-oriented nanomedicine framework, this represents an important intermediate observation, as it supports further evaluation of the formulation in more complex tumor-mimetic models. This interpretation is also consistent with broader nanomedicine literature in prostate cancer, which emphasizes that advanced delivery systems should be evaluated not only for molecular potency but also for their ability to retain activity in more complex tumor-mimetic environments^39^. In addition, HA-coated apigenin-loaded nanoparticle systems have shown that receptor-relevant carrier design may preserve or enhance antitumor performance beyond conventional monolayer settings, even across different tumor models^40^.

The preliminary ARPE19 data also deserve balanced interpretation. The selected lower-dose SeNP-apigenin combination showed the most acceptable tolerability profile among the tested formulations, which supported its use as the working condition in the mechanistic assays. At the same time, the reduction in ARPE19 viability remained statistically significant relative to the untreated control. Importantly, these ARPE19 data were derived from a single non-tumoral cell line and should not be interpreted as a safety assessment or as evidence of a defined therapeutic index. Accordingly, the present data should not be framed as proof of safety, but rather as an initial indication that lower-dose loading offers a comparatively more favorable balance between antitumor activity and off-target toxicity than higher-dose combinations. This nuance is important because it avoids overinterpretation while still supporting the view that the formulation represents a rational candidate for further refinement. Recent studies on selenium-based nanotherapeutics likewise indicate that optimization of dose, loading, and carrier architecture is critical for improving therapeutic index while minimizing unintended effects on non-tumoral cells^41^.

Several limitations should also be acknowledged. First, the biological conclusions are restricted to a LNCaP cell model and should not be generalized to all prostate cancer phenotypes. Second, although HA coating was associated with improved functional performance, the study was not designed to directly prove receptor-mediated uptake or CD44-dependent selectivity. Third, while the 3D spheroid results strengthen the biological relevance of the findings, in vivo validation is required before stronger translational claims can be made. Fourth, several mechanistic layers were not directly assessed, including PI3K/AKT phosphorylation, AR protein abundance and nuclear localization, AR recruitment to PSA regulatory regions, cleaved caspase/PARP activation, BAX/BCL2 protein ratio, and protein-level validation of SOX2 or OCT3/4. Therefore, the mechanistic interpretation remains restricted to transcript-level, ELISA-based PSA, flow cytometric, viability, and spheroid readouts. Fifth, HA-coating stability and apigenin release kinetics were not evaluated in complex media mimicking the prostatic interstitial environment; therefore, sustained AR/PSA-axis modulation cannot be inferred from release behavior in the present study. Finally, the ARPE19 assay provides only preliminary tolerability information and does not replace broader safety evaluation. These limitations define the appropriate boundaries of interpretation and highlight the need for further pharmacokinetic, mechanistic, and in vivo validation before stronger therapeutic claims can be justified^42–44^.

In conclusion, the present findings support further preclinical evaluation of this nanoparticle-based formulation in which apigenin provides the core natural-product component, while selenium nanoparticle engineering and hyaluronic acid coating were associated with a stronger biological response across the measured endpoints under the tested in vitro conditions. In androgen-responsive prostate cancer cells, HA-SeNP-Api attenuated measured AR/PSA-associated readouts, increased Annexin V/PI-defined apoptotic populations, was associated with checkpoint-related transcriptional changes, and reduced selected plasticity-associated transcripts. Taken together, these results suggest that the formulation may represent a candidate nanoformulation for further investigation in androgen-responsive prostate cancer models, although further studies are required to clarify uptake mechanisms, broader selectivity, and in vivo performance.

## Methods

### Preparation of HA-SeNP-Api

Hyaluronic acid-coated, apigenin-loaded selenium nanoparticles (HA-SeNP-Api) were prepared using a reduction-based chemical method adapted from previously reported selenium nanoparticle synthesis procedures^40,45–47^ and in accordance with the formulation strategy previously established for the same nanoparticle platform. Briefly, sodium selenite and L-ascorbic acid solutions were mixed under controlled stirring conditions to generate selenium nanoparticles (SeNPs). After nanoparticle formation, apigenin was added to the SeNP suspension and incubated to allow drug loading, followed by dropwise addition of hyaluronic acid solution to obtain the HA-coated formulation. Detailed physicochemical characterization of the HA-SeNP-Api formulation, including particle size, zeta potential, encapsulation efficiency, and in vitro release profile, has been reported in our companion study^27^. To improve readability and provide formulation context without duplicating the companion study, a concise summary of these previously reported physicochemical properties is provided in Supplementary Table S2.

### Cell culture conditions

The human prostate cancer cell line LNCaP was used as the experimental model^48^. The LNCaP cell line was obtained from ATCC (Manassas, VA, USA). Cells were maintained in Dulbecco’s Modified Eagle Medium (DMEM) supplemented with 10% fetal bovine serum, 1% penicillin-streptomycin, and 2 mM L-glutamine. Cultures were incubated at 37 °C in a humidified atmosphere containing 5% CO₂. Cells were passaged under standard sterile conditions and used after reaching the appropriate confluence for each assay.

### Cytotoxicity analysis by MTT assay

Cytotoxicity was evaluated using the MTT assay. LNCaP cells were seeded into 96-well plates at a density of 1 × 10⁴ cells/well and incubated for 24 h. Cells were then exposed to the indicated agents in culture medium for 72 h. At the end of the treatment period, the medium was removed, the cells were washed with phosphate-buffered saline (PBS), and 1 mg/mL MTT solution (Sigma-Aldrich) was added to each well. Following 3 h of incubation, the resulting formazan crystals were dissolved in dimethyl sulfoxide (DMSO), and absorbance was measured at 570 nm using a Multiskan GO microplate reader (Thermo Fisher Scientific). Cell viability was calculated according to the following formula: viability (%) = (OD_sample / OD_control) × 100.

To identify a biologically active sub-IC_50_ concentration of apigenin for subsequent mechanistic experiments, LNCaP cells were treated for 72 h with apigenin at concentrations of 3.12, 6.25, 12.5, and 25 µM, and viability was assessed by MTT assay. Based on the resulting viability profile, 6.25 µM was selected as the working sub-IC_50_ concentration for downstream analyses.

To compare formulation-dependent effects, cells were additionally treated with ascorbic acid alone (AA, 125 µM), selenium nanoparticles alone (SeNP, 100 µM), apigenin-loaded SeNPs without HA coating [HA(−)], or hyaluronic acid-coated apigenin-loaded SeNPs [HA(+)]. The HA(−) formulation consisted of 100 µM SeNP + 6.25 µM apigenin without HA coating, whereas HA(+) corresponded to the HA-coated formulation containing 100 µM SeNP + 6.25 µM apigenin.

To obtain a preliminary indication of tolerability in a non-tumoral model, ARPE19 cells were exposed to selected SeNP-apigenin combinations consisting of 100 µM SeNP + 6.25 µM apigenin, 100 µM SeNP + 25 µM apigenin, 200 µM SeNP + 6.25 µM apigenin, and 200 µM SeNP + 25 µM apigenin. Cell viability in all groups was expressed relative to the untreated control.

Enzalutamide was tested at 6.25 µM, matching the sub-IC_50_ apigenin concentration selected for this study. This concentration was not intended to represent a clinically equivalent exposure, but rather to allow a mechanistically informative comparison of transcriptional and apoptotic responses under equivalent dosing conditions within the same experimental model.

### RNA isolation and RT-qPCR analysis

Following treatment, total RNA was isolated using the EZ-10 Spin Column Total RNA Mini-Preps Kit (Bio Basic, Canada; BS1361/SK8655) according to the manufacturer’s instructions. Briefly, cells were lysed, RNA was purified using a silica membrane spin-column system, contaminants were removed by sequential wash steps, and purified RNA was eluted in elution buffer. RNA quantity and purity were assessed prior to downstream applications.

Complementary DNA (cDNA) was synthesized from isolated RNA using the OneScript Plus cDNA Synthesis Kit (ABMgood, G236) according to the manufacturer’s recommended protocol. Equal amounts of RNA were used in each reverse transcription reaction. Real-time quantitative PCR (RT-qPCR) was performed using BlasTaq 2X qPCR MasterMix (ABMgood, G891), and amplifications were carried out on a Bio-Rad CFX96 real-time PCR system (Bio-Rad, USA). GAPDH was used as the internal reference gene, and relative gene expression levels were calculated using the 2 − ΔΔCt method^49^.

The gene panel included AR and PSA as androgen-axis markers; p53, p21, p27, BAX, BCL2, CASP3, CASP8, APAF1, survivin, and TNFR1 to evaluate apoptosis- and checkpoint-related responses; and CD44, NANOG, SOX2, OCT3/4, and SLUG to assess stemness/plasticity-associated transcriptional programs. All RT-qPCR experiments were performed using three independent biological replicates, and each biological replicate was analyzed in technical triplicate. Primer sequences used for RT-qPCR are provided in Supplementary Table S1.

### PSA protein analysis by ELISA

PSA protein levels were determined using a commercial sandwich ELISA kit (Sunred, Human Prostate Specific Antigen [PSA] ELISA Kit, Cat. No. 201-12-1714, 96 tests) according to the manufacturer’s instructions. LNCaP cells were treated with the indicated agents for 72 h, after which conditioned culture media were collected and processed according to the assay protocol. Absorbance was measured at 450 nm using a microplate reader, and PSA levels were expressed relative to the control group.

### Apoptosis analysis

Apoptosis levels were determined by Annexin V-FITC/propidium iodide (PI) staining^50^. LNCaP cells were seeded into 6-well plates at a density of 2.5 × 10⁵ cells/well and treated with the indicated agents for 72 h. After treatment, cells were trypsinized, washed twice with cold PBS, and resuspended in Annexin binding buffer. Cells were incubated with Annexin V-FITC for 15 min in the dark, followed by PI staining, and analyzed using a BD FACSCalibur flow cytometer (BD Biosciences, USA). Total apoptosis was calculated as the sum of early and late apoptotic cell populations.

### Cell-cycle analysis

Cell-cycle distribution was evaluated by PI staining. LNCaP cells were seeded into 6-well plates at an appropriate density and treated with the indicated agents for 72 h. After treatment, cells were collected, fixed overnight in 70% ethanol at 4 °C, and then resuspended in PBS containing RNase A (100 µg/mL) and PI (50 µg/mL). Samples were incubated for 30 min in the dark and analyzed using a BD FACSCalibur flow cytometer (BD Biosciences, USA). Cell-cycle phase distribution was evaluated as the percentage of cells in G0/G1, S, and G2/M phases.

### Three-dimensional spheroid culture and analysis

To assess treatment responses under three-dimensional culture conditions, 96-well plates coated with agarose (Nest Scientific) were used for spheroid formation. To prevent cell attachment, the bottom of each well was coated with 70 µL of 1.5% agarose solution (Sigma-Aldrich). After solidification of the agarose layer, LNCaP cells were seeded at a density of 2 × 10³ cells/well in 100 µL culture medium. Under these non-adherent conditions, cells were unable to attach to the plate surface and instead aggregated to form spheroidal structures. The cultures were subsequently treated with the defined experimental groups for the designated treatment period. Spheroid morphology and growth behavior were documented microscopically, and treatment-related responses were evaluated by comparing spheroid integrity, expansion, and final cellular burden across groups.

### Statistical analysis

All data were obtained from at least three independent biological replicates. For RT-qPCR analyses, each biological replicate was measured in technical triplicate, and the mean of the technical replicates was used for statistical evaluation. Data were first assessed for suitability for parametric analysis, including homogeneity of variance. For datasets showing unequal variances, Brown-Forsythe and Welch ANOVA followed by Games-Howell or Dunnett’s T3 multiple-comparisons tests were used when appropriate. For datasets meeting parametric assumptions, one-way ANOVA followed by Tukey’s post hoc test was applied. Data are presented as mean ± SEM, and *P* < 0.05 was considered statistically significant. For spheroid diameter data obtained from the same spheroids at Day 0, Day 5, and Day 10, two-way repeated measures ANOVA with Geisser–Greenhouse correction was used, followed by Tukey’s multiple-comparisons test for simple effects within each time point. Final spheroid cell counts were analyzed using one-way ANOVA followed by Tukey’s post hoc test.

### AI-assisted language editing

During manuscript preparation, ChatGPT (OpenAI) was used solely for language editing and improvement of readability. No artificial intelligence tool was used for data analysis, data interpretation, or generation of scientific conclusions. All scientific content, interpretations, and final decisions were made by the authors.

## Supplementary Information

Below is the link to the electronic supplementary material.


Supplementary Material 1


## Data Availability

The datasets generated and/or analyzed during the current study are available from the corresponding author on reasonable request.
