# Supplementary material for: Nanoparticle-enhanced apigenin suppresses androgen receptor signaling and cellular plasticity in LNCaP cells
Source: Sci Rep. 2026 Jul 23;16:27273. doi: 10.1038/s41598-026-63769-5 (PMC13529782; doi:10.1038/s41598-026-63769-5)
Supplement: Supplementary file 1 [file 41598_2026_63769_MOESM1_ESM.docx]

**Supplementary Information**

Nanoparticle-enhanced apigenin suppresses androgen receptor signaling and cellular plasticity in LNCaP cells

Sinan Vicil, Rıza Serttas, Suat Erdogan

**Supplementary Figure S1. Proposed formulation assembly and cell-associated interaction of HA-SeNP-Api in LNCaP cells.**

**(A)** Stepwise assembly of the HA-SeNP-Api nanosystem through selenium nanoparticle formation, apigenin loading, and hyaluronic acid coating.

**(B)** Proposed cell-associated interaction of HA-SeNP-Api in LNCaP cells. This schematic is conceptual and does not demonstrate receptor-mediated uptake.

**(C)** Main biological readouts evaluated in the study, including reduced AR/PSA readouts, increased apoptosis-associated response, altered cell-cycle distribution, reduced plasticity-associated transcripts, and reduced 3D spheroid growth.

**Supplementary Table S1. Forward and reverse primer sequences (5′–3′) used for quantitative real-time PCR (RT-qPCR) analysis**

| **Gene** | **Forward primer (5′–3′)** | **Reverse primer (5′–3′)** |
| --- | --- | --- |
| ***AR*** | ATGTCCAGGATCTGGAGAAG | GCAGAGTGAGGAGGATGGA |
| ***PSA*** | ACCAGAGGAGTTCTTGACCCCAAA | CCCCAGAATCACCCGAGCAG |
| ***CD44*** | TCCCAGACGAAGACAGTCCCTGGAT | CACTGGGGTGGAATGTGTCTTGGTC |
| ***SOX2*** | GCCGAGTGGAAACTTTTGTCG | GCAGCGTGTACTTATCCTTCTT |
| ***OCT3/4*** | ACATGTGTAAGCTGCGGCC | GTTGTGCATAGTCGCTGCTTG |
| ***NANOG*** | ATGCCTCACACGGAGACTGT | AGGGCTGTCCTGAATAAGCA |
| ***BAX*** | TTGCTTCAGGGTTTCATCCA | CAGCCTTGAGCACCAGTTTG |
| ***BCL2*** | ATGTGTGTGGAGAGCGTCAA | ACAGTTCCACAAAGGCATCC |
| ***CASP3*** | GGCATTGAGACAGACAGTGG | CATGGAATCTGTTTCTTTGC |
| ***CASP8*** | CTGCTGGGGATGGCCACTGTG | TCGCCTCGAGGACATCGCTCTC |
| ***APAF1*** | TGGTCAACTGCAAGGACCAT | CACGTTCAAAGGTGGCTGAT |
| ***SLUG*** | CAACCCACTCAGATGTCAA | CATAGTTAGTCACACCTCGT |
| ***TNFR1*** | AGGGGACAGGGAGAAGAGAG | GTGCACACGGTGTTCTGTTT |
| ***Survivin*** | GACGACCCCATAGAGGAACA | GACAGAAAGGAAAGCGCAAC |
| ***P53*** | GAGGTTGGCTCTGACTGTACC | TCCGTCCCAGTAGATTACCAC |
| ***P21*** | GGCGTTTGGAGTGGTAGAAA | GACTCTCAGGGTCGAAAACG |
| ***P27*** | CCGGCTAACTCTGAGGACAC | TTGCAGGTCGCTTCCTTATT |
| ***Cyclin B*** | GCACTTTCCTCCTCTCA | TTCGATGTGGCATACTTG |
| ***GAPDH*** | TTGGTATCGTGGAAGGACTCA | TGTCATCATATTTGGCAGGTTT |

**Supplementary Table S2. Summary of previously reported physicochemical properties of the HA-SeNP-Api formulation from the companion study.**

| **Parameter** | **Reported value / observation** | **Method / condition** |
| --- | --- | --- |
| Hydrodynamic size | 169.80 ± 4.45 nm | DLS, PBS pH 7.4, 0 h |
| Hydrodynamic size | 263.90 ± 3.72 nm | DLS, PBS pH 7.4, 72 h |
| Hydrodynamic size | 223.60 ± 5.21 nm | DLS, acidic PBS pH 6.0, 0 h |
| Hydrodynamic size | 231.80 ± 3.97 nm | DLS, acidic PBS pH 6.0, 72 h |
| Hydrodynamic size | 192.40 ± 3.75 nm | DLS, PBS + 10% FBS, 0 h |
| Hydrodynamic size | 542.50 ± 32.00 nm | DLS, PBS + 10% FBS, 72 h |
| PDI | 0.45 ± 0.02 | PBS pH 7.4, 0 h and 72 h |
| PDI | 0.25 ± 0.01 to 0.54 ± 0.02 | Acidic PBS pH 6.0, 0 h to 72 h |
| PDI | 0.32 ± 0.02 to 0.51 ± 0.02 | PBS + 10% FBS, 0 h to 72 h |
| Zeta potential | −27.8 ± 0.8 mV | PBS pH 7.4, 0 h |
| Zeta potential | −26.83 ± 1.40 mV | PBS pH 7.4, 72 h |
| Zeta potential | −4.2 ± 0.9 to −1.2 ± 1.08 mV | Acidic PBS pH 6.0, 0 h to 72 h |
| Zeta potential | −12.1 ± 0.8 to −13.5 ± 1.1 mV | PBS + 10% FBS, 0 h to 72 h |
| Encapsulation efficiency | 94.2% | LC-MS/MS |
| Release profile | ~89–93% cumulative release at 72 h | PBS pH 6.0 and pH 7.4 |
| Release profile | ~66–67% cumulative release at 72 h | PBS + 10% FBS |
| Morphology | Predominantly spherical/subspherical particles; partial aggregation | SEM |

Values are summarized from the companion study by Vicil et al.^27^ and are provided only to improve readability and formulation context. These physicochemical characterization data were not newly generated in the present study.
